# Congenital Anomalies of the Left Main Coronary Artery: A Case Series

**DOI:** 10.7759/cureus.62663

**Published:** 2024-06-19

**Authors:** Edvin Dado, Hortensia Gjergo, Ervina Shirka, Leonard Simoni, Augusta Gjika

**Affiliations:** 1 Department of Cardiology, University Hospital Center "Mother Teresa", Tirana, ALB

**Keywords:** coronary ostium, congenital anomalies of coronary arteries, anomalous left main coronary artery, cadiothoracic surgery, computed tomographic angiography

## Abstract

Coronary artery anomalies (CAAs) are rare congenital defects. The most frequent congenital anomaly is the origin of the left circumflex artery (LCX) from the right coronary sinus, followed by the common origin of the right coronary artery (RCA) and left anterior descending artery (LAD) from the right coronary sinus, as well as LAD originating from the right coronary sinus. The rarest anomaly is the left coronary artery or left main (LM) originating from the right coronary sinus. CAAs can occur with various anatomical features according to their origin and course. In this article, we will present four cases with congenital anomalies of the LM according to the above variations detected through coronary CT angiogram (CCTA) and coronary angiography. These cases involve an LM coronary artery originating from the right coronary sinus and pre-aortic course, retro-aortic course, LM originating from the sino-tubular junction above the left coronary sinus, and complete absence of LM. The incidence of these cases is rare, so their diagnosis is very important for patients' management and follow-up.

## Introduction

Coronary artery anomalies (CAAs) are rare congenital defects affecting 0.3-1% of the population [1]. However, their incidence varies widely and is not yet determined, so it remains the subject of numerous studies. Congenital anomalies may remain asymptomatic throughout an individual's life; however, in certain cases, they are hemodynamically significant. Clinical manifestations in adults vary from typical chest pain, symptoms of heart failure, and acute myocardial infarction to sudden death even at a young age [2]. Congenital anomalies of the coronary arteries can be incidentally discovered during coronary CT angiogram (CCTA) and coronary angiography in patients with chest pain. Angiography remains the gold standard for the diagnosis and intraluminal assessment of the coronary arteries; however, this procedure fails to provide a three-dimensional assessment of vessel trajectories and their relationship with adjacent structures. In these cases, CCTA and magnetic resonance imaging (MRI) are highly valuable, providing a comprehensive assessment of coronary artery course and relationships with major vessels [3,4]. The anomalous origin of the left main (LM) coronary trunk is a rare condition and might be associated with an increased risk of cardiac events. Knowing its anomalies is very important as it may pose a lot of difficulties in management and revascularization strategies. Therefore, we present in this article different uncommon cases of LM anomalies that differ in their clinical and imaging presentation.

## Case presentation

Case 1

LM Originating from the Right Coronary Sinus with Pre-aortic Course

**Figure 1:**
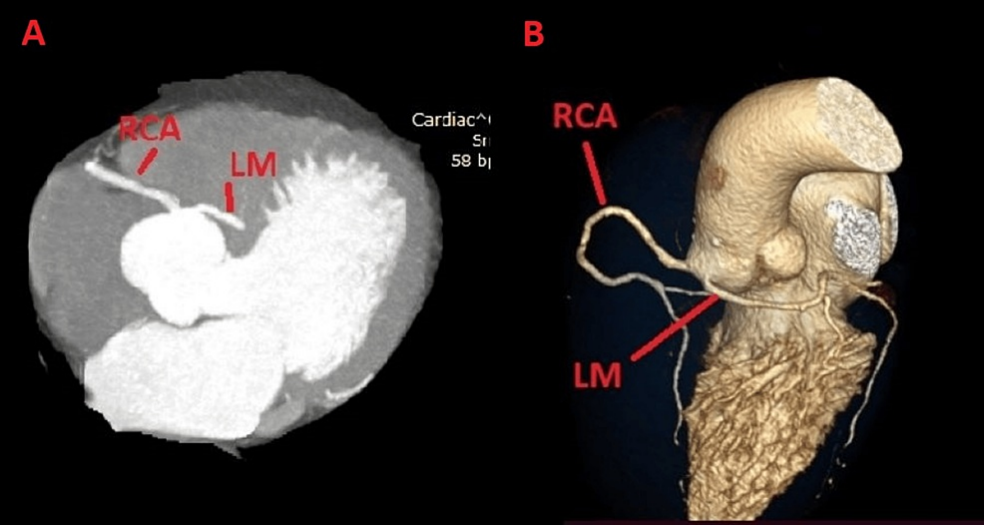
Case 1 A) Two-dimensional coronary CTA image showing LM originating from the right coronary sinus with a distinct ostium from RCA. B) Three-dimensional coronary CTA image showing LM originating from the right coronary sinus with a distinct ostium from RCA with pre-aortic course. CCTA: Coronary CT Angiogram; RCA: Right Coronary Artery; LM: Left Main

A 69-year-old male presents to the ED with retrosternal chest pain and high blood pressure. The patient was hypertensive and a non-smoker, and he had a family history of coronary artery disease. On physical examination, rhythmic heart sounds were noted with a heart rate of 65 beats per minute (bpm), without pathological murmurs. Blood pressure was 170/90 mmHg. Bilateral vesicular breath sounds with oxygen saturation (SpO_2_) of 99% were observed. The rest of the physical examination was within normal limits. An ECG showed sinus rhythm with negative T waves in lead III. Biochemical analysis parameters were within normal limits, including negative cardiac enzymes. Echocardiography revealed left ventricular hypertrophy with normal cardiac function. The patient was referred for CCTA, which revealed an anomaly in the origin of the coronary arteries, specifically: LM originating from the right coronary sinus with a distinct ostium from the right coronary artery (RCA) and a pre-aortic course without intraluminal stenosis, as seen in Figure 1. The patient was discharged from our hospital with reinforcement of antihypertensive therapy. He had no symptoms at the six-month follow-up.

Case 2

LM Originating from the Right Coronary Sinus with Retro-aortic Course

**Figure 2:**
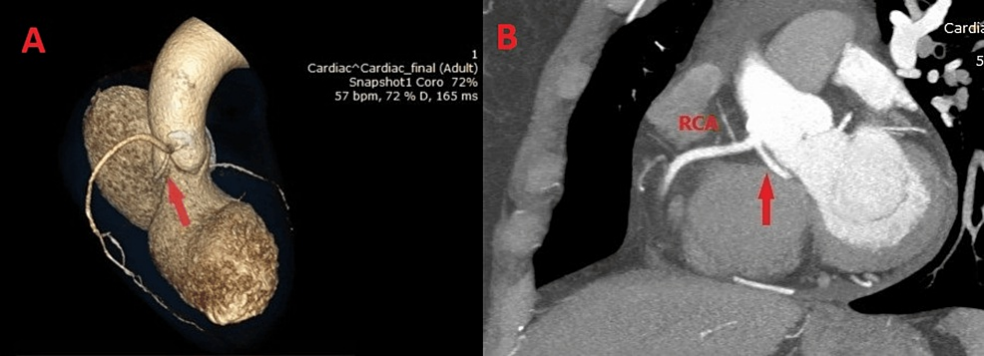
Case 2 A) Three-dimensional CCTA image showing LM (red arrow) originating from the right coronary sinus with retro-aortic course. B) Two-dimensional CCTA image showing LM (red arrow) originating from the right coronary sinus with retro-aortic course. CCTA: Coronary CT Angiogram; LM: Left Main

A 54-year-old female presents to the ED with retrosternal chest pain. She reports a several-month history of chest pain with palpitations and mild dyspnea. She is known to have hypertension and dyslipidemia. On physical examination, she presents with rhythmic heart sounds without pathological murmurs, with a heart rate of 72 bpm. On lung examination, bilateral vesicular breath sounds with SpO_2_ of 98% were noted. The ECG showed a sinus rhythm without specific ST-T changes, with the presence of an atrial premature ectopic beat. An echocardiography revealed left ventricular hypertrophy and mild mitral regurgitation. The patient underwent a stress test, which was inconclusive for coronary artery disease, with an exercise capacity of 6.6 metabolic equivalent (MET). Therefore, CCTA was recommended, revealing a coronary anomaly, LM originating from the right coronary sinus with the retro-aortic course, as seen in Figure 2. Coronary arteries showed minimal calcification without significant stenosis. The patient was recommended for conservative treatment with reinforcement of antihypertensive and antilipemic treatment. She did not have any additional symptoms in the subsequent follow-up.

Case 3

Absence of LM: Left Anterior Descending Artery (LAD), Left Circumflex Artery (LCX), and Right Coronary Artery (RCA) Originating from the Right Coronary Sinus

**Figure 3:**
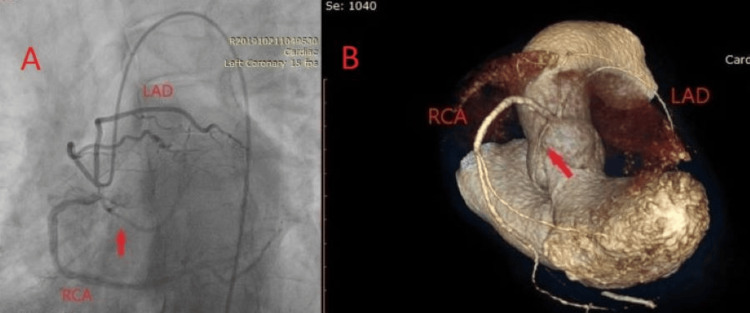
Case 3 A) Coronary angiography view showing LAD, RCA, and LCX originating from the right coronary sinus. The arrow indicates 50% stenosis of LCX. B) CCTA image showing RCA with a normal course, LAD originating from the right coronary sinus, while LCX (red arrow) has a retro-aortic course. CCTA: Coronary CT Angiogram; LAD: Left Anterior Descending Artery; RCA: Right Coronary Artery; LCX: Left Circumflex Artery

A 58-year-old male presents to the ED with retrosternal chest pain for the first time, lasting about 30 minutes, and dyspnea. He had no significant past medical history. On admission, his vital signs indicated a regular pulse, blood pressure of 160/70 mmHg, and oxygen saturation of 98%. The physical examination revealed a systolic murmur at the cardiac apex and bilateral vesicular breath sounds. The ECG showed a sinus rhythm with a heart rate of 70 bpm and signs of left ventricular hypertrophy. Biochemical analyses showed alterations in blood glucose levels and slightly elevated creatinine kinase (CK) and CK-MB values with negative high-sensitivity troponin levels. We performed coronary angiography that revealed the abnormal origin of LAD, LCX, and RCA from the right coronary sinus and a single vessel disease with 50% stenosis in the LCX artery, as seen in Figure 3A. To assess the relationship of coronary arteries with major vessels, CCTA was performed, confirming the absence of LM and the coronary arteries originating from a common ostium in the right coronary sinus. The LAD courses are anterior to the pulmonary trunk, while LCX had a retro-aortic course, as seen in Figure 3B. Because of the complexity of this anomaly, the patient was managed conservatively with beta-blockers, vasodilators, and antilipemic drugs. At the three-month follow-up, the patient was in stable condition.

Case 4

LM Originating from the Sino-tubular Junction Above the Left Coronary Sinus

**Figure 4:**
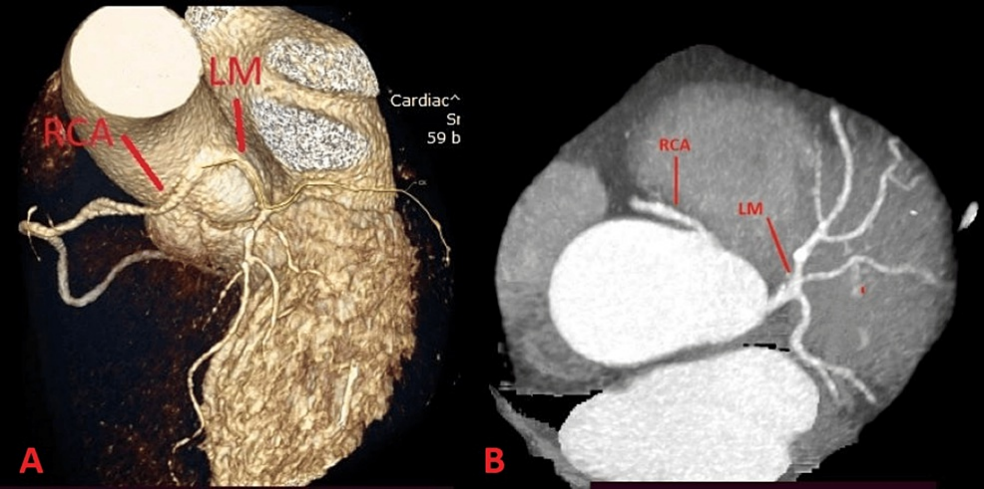
Case 4 A) Three-dimensional CCTA image showing LM and RCA originating from the sino-tubular junction above the left coronary sinus. B) Two-dimensional CCTA image showing LM originating from the sino-tubular junction and RCA with an interarterial course between the aortic bulb and the pulmonary trunk. CCTA: Coronary CT Angiogram; RCA: Right Coronary Artery; LM: Left Main

A 70-year-old male presented to the ED with a typical angina. He reported a history of chest pain during moderate physical exertion for several months. The patient had a history of hypertension and smoking for about 10 years. On physical examination, rhythmic heart sounds without pathological murmurs, a heart rate of 77 bpm, and bilateral vesicular breath sounds with SpO_2_ of 98% were noted. The ECG showed sinus rhythm without specific ST-T changes. Echocardiography revealed a left ventricle with normal dimensions and ejection fraction. Biochemical analyses showed negative cardiac enzymes. CCTA revealed CAA. The LM and RCA originated from the sino-tubular junction above the left coronary sinus, and RCA had an interarterial course between the aortic bulb and the pulmonary trunk (Figure 4). The CT detected LAD severe stenosis, which was then confirmed on coronary angiography. Considering the presence of coronary anomaly and the LAD severe stenosis, the patient was referred to cardiac surgery. He underwent coronary artery bypass and had an uneventful postoperative hospital course.

## Discussion

According to the literature, congenital anomalies of the coronary arteries affect about 1% of the general population, ranging from 0.3% to 5.6% in studies on patients undergoing coronary angiography and routine autopsies [5]. The incidence of LM coronary anomaly ranges between 0.02% and 0.07% [6]. In 0.41% of cases, the LM artery is absent. In this condition, the LAD and LCX bifurcate separately. The LM CAAs might include anomalies of the origin where the left coronary artery may arise from an inverted coronary sinus such as the right coronary sinus or from another vessel such as the pulmonary artery. The latter may lead to myocardial ischemia and even heart failure if not corrected. The second anomaly variation might be anomalies of course where the left coronary artery may take an abnormal course, such as passing between the aorta and pulmonary artery (interarterial course). This anomaly may result in compression of the coronary artery between these two structures during exertion or tachycardia, leading to episodes of myocardial ischemia up to sudden death. In our cases, we saw two different courses of LM: retro-aortic and pre-aortic courses. The last group of variations is coronary fistulas. These are anomalies in the termination of the coronary arteries, such as abnormal termination in the left ventricle or atrium, leading to inadequate coronary perfusion, ischemia, or myocardial volume overload.

Congenital anomalies may remain asymptomatic throughout an individual's life, but in certain cases, they are hemodynamically significant. Congenital anomalies of the LM are relatively rare but can have significant clinical implications. Clinical manifestations in adults vary from asymptomatic cases to symptoms of myocardial ischemia or sudden death.

The gold standard for detecting coronary anomalies and intraluminal stenosis is coronary angiography. However, this method provides only two-dimensional views of the coronary artery course; hence, the relationship of the vessel with the aorta and pulmonary artery may be difficult to differentiate. Moreover, during the coronary angiography procedure, the artery with an anomalous origin may be difficult to cannulate, not being in its standard anatomical position. In such cases, this may be misinterpreted as an occluded vessel. For this reason, new imaging methods such as CCTA and MRI are crucial for the diagnosis. The use of CCTA creates opportunities for three-dimensional views to accurately identify the origin and course of the coronary arteries and their relationships with nearby structures or associated congenital anomalies [7].

Symptomatic patients with CAAs have three treatment options: medical treatment/observation, percutaneous coronary intervention (PCI), and surgical treatment. Medical treatment mainly comprises beta-blocker therapy and lifestyle modifications. PCI is recommended in patients with atherosclerotic stenosis above 75% and in patients who exhibit clinically reversible ischemic symptoms or documented ischemia by functional tests. Large-scale studies regarding the experience and data on surgical treatment are lacking. Generally, this option is recommended in anomalies of the origin of the coronary artery from an inverse sinus, when a large myocardial territory is at risk of ischemia and PCI is not feasible [8].

## Conclusions

LM artery anomalies remain among rare congenital defects. The recognition of these coronary anomalies is very important to ensure accurate angiographic interpretation, and it is crucial for the final treatment decision of patients with ischemia. This article aims to present different clinical and imaging variations of the LM congenital anomaly. The identification of such cases improves further acknowledgment of the variations, diagnosis, and treatment of coronary anomalies.
